# ctDNA in Pancreatic Adenocarcinoma: A Critical Appraisal

**DOI:** 10.3390/curroncol32110589

**Published:** 2025-10-22

**Authors:** Sujata Ojha, William Sessions, Yuhang Zhou, Kyaw L. Aung

**Affiliations:** 1 Department of Internal Medicine, Dell Medical School, University of Texas at Austin, Austin, TX 78712, USA; sujata.ojha@austin.utexas.edu; 2 Division of Hematology and Oncology, Dell Medical School, University of Texas at Austin, Austin, TX 78712, USA; william.sessions@ascension.org (W.S.); yuhang.zhou@ascension.org (Y.Z.)

**Keywords:** circulating tumor DNA (ctDNA), pancreatic ductal adenocarcinoma (PDAC), minimal residual disease (MRD), precision oncology, liquid biopsy

## Abstract

**Simple Summary:**

Detection of cancer derived mutations in blood could offer a more accurate assessment of patient prognosis, their response to therapies, and a better way to monitor the biology of the patients’ cancer and select more appropriate treatments for individual patients. However, despite the promise and progress in the field of testing circulating tumor DNA (ctDNA) in patients with cancers, concrete evidence that shows that ctDNA testing benefits the clinical outcomes of patients with pancreatic ductal adenocarcinoma (PDAC) is still lacking. More rigorous validation studies within specific clinical contexts are needed before the true potential of ctDNA can be realized for patients with PDAC.

**Abstract:**

Pancreatic ductal adenocarcinoma (PDAC) is one of the deadliest malignancies due to late diagnosis and limited treatment options. Circulating tumor DNA (ctDNA) is a promising, minimally invasive biomarker that could improve the clinical outcomes of patients with PDAC by enabling early disease detection, minimal residual disease (MRD) assessment, precise prognostication, and accurate treatment monitoring. CtDNA has prognostic as well as predictive value in both resectable and metastatic settings, with serial measurements enhancing risk stratification and recurrence prediction beyond CA19-9. However, despite the promise, the true potential of ctDNA has not yet been fulfilled in patients with PDAC. The current limitations include a low sensitivity of ctDNA assays in early stage PDAC, challenges in the assay interpretation due to the specific nature of ctDNA shedding in PDAC, inter-patient heterogeneity, and technical variability. As precision oncology advances, ctDNA will be a powerful tool for personalized care in PDAC, but rigorous validation of its use within specific clinical contexts is still needed before the true potential of ctDNA is realized for patients with PDAC.

## 1. Introduction

Pancreatic ductal adenocarcinoma (PDAC) is among the deadliest malignancies worldwide, with a 5-year survival rate of less than 15% [1]. Surgical resection is the only curative treatment option, but most patients present late with locally advanced unresectable disease or metastatic disease. Although there are well-recognized risk factors for the development of PDAC, including inherited pathogenic mutations in cancer susceptibility genes such as *STK11* and *BRCA1/2*, chronic diseases such as chronic pancreatitis and diabetes, and lifestyle factors such as tobacco (15–30% of cases) and alcohol use, currently, existing biomarkers like CA19-9 and imaging modalities lack sufficient sensitivity for early stage detection or precise dynamic disease monitoring [2,3]. Thus, new improved disease specific markers are needed for early detection and making better clinical decisions.

Recently, circulating tumor DNA (ctDNA), a subset of cell-free DNA (cfDNA), has emerged as a promising biomarker that offers real-time, noninvasive insights into disease burden, treatment response, and disease recurrence [4,5,6,7]. First identified in the 1970s, the technological advances made in recent decades, including next-generation sequencing (NGS), digital PCR, and tumor-informed assays, have dramatically improved ctDNA detection [8,9,10]. ctDNA, a cornerstone of liquid biopsy, has now emerged as a crucial biomarker for the detection of minimal residual disease (MRD) and recurrence risk stratification in solid tumors, with the most robust evidence shown to date in colorectal cancer (CRC) [6]. Recent improvements in assay sensitivity and the integration of artificial intelligence (AI) have further broadened ctDNA applications in early detection and treatment planning [11,12]. In 2020, the U.S. Food and Drug Administration (FDA) approved ctDNA-based liquid biopsies to detect actionable mutations such as pathogenic mutations in *BRCA1* and *BRCA 2* in patients with ovarian and prostate cancers, *ALK* rearrangement in non-small cell lung cancer, and *PIK3CA* mutations in breast cancer, with Medicare coverage implemented since 2021. These ctDNA based tests currently guide treatment decisions in oncology clinics.

Compared with tissue biopsy, liquid biopsy is less invasive, associated with fewer complications, and offers faster turnaround times. As NGS becomes more accessible and affordable, ctDNA-based liquid biopsies are expected to expand into broader clinical applications in oncology. However, despite all the advances we have seen in the ctDNA research field, there is currently no FDA approved ctDNA-based assay for use in patients with PDAC. Thus, perhaps the time is ripe for a critical appraisal of the status of ctDNA as a biomarker in this disease. This review summarizes key aspects of ctDNA clinical applications across PDAC stages, offering oncologists, researchers, and trainees an evidence-based appraisal.

## 2. Current Evidence on ctDNA Clinical Utility in PDAC

### 2.1. ctDNA as a Novel Prognostic Marker in Advanced PDAC

In patients with advanced metastatic disease, ctDNA has robust data supporting its use as a prognostic biomarker. Multiple prospective, retrospective studies, and metanalyses have demonstrated that the presence of detectable ctDNA at the baseline in patients with metastatic PDAC is associated with shorter overall survival (OS) and progression free survival (PFS) compared with ctDNA negative patients, even after adjusting for tumor burden, CA19-9 levels, and other risk factors [13,14,15,16,17,18]. Thus, ctDNA is an independent prognostic biomarker in advanced PDAC.

Previous studies have found that ctDNA and CA 19-9, the only FDA approved tumor marker in PDAC, are positively correlated and offer complementary prognostic information [15,16,19,20]. High CA19-9 levels are associated with an increased likelihood of ctDNA detection, and both markers are independently associated with worse survival outcomes [20,21,22,23]. In a large cohort of unresectable PDAC patients, the baseline ctDNA and CA19-9 correlated positively, however, remained independently associated with time to progression along with OS in multivariate analysis [20]. Additionally, ctDNA was detectable in some patients without CA 19-9 elevation, underscoring its non-overlapping utility. In a large colorectal cancer cohort, Botta et al. demonstrated that personalized ctDNA assays outperformed traditional tumor markers in predicting recurrence and remained independently prognostic even after adjusting for CA 19-9 and CEA [24]. While not all studies are PDAC-specific, these findings support the potential for ctDNA to complement or surpass CA19-9 in prognostic utility [24]. Similarly, a prospective ancillary study of the PANACHE01-PRODIGE48 trial found that both high CA19-9 and ctDNA positivity at the time of diagnosis were independently associated with worse OS. When combined, the biomarkers have superior risk stratification compared with either alone, with the worst outcomes in patients positive for both [25].

The diagnostic accuracy of CA19-9 and ctDNA depends on the stage of disease and the type of assay. CA19-9 remains the most used biomarker but is limited by false positives in benign disease and false negatives in Lewis antigen-negative patients. On the other hand, ctDNA offers higher specificity, but lower sensitivity in early stage disease. Table 1 summarizes the evidence behind the sensitivity and specificity of these biomarkers. The potential of ctDNA as a screening tool and for early detection is discussed in the next section.

### 2.2. ctDNA as a Tool for PDAC Screening/Early Detection

PDAC is deadly as it is notoriously difficult to diagnose in its early stages due to the absence of specific symptoms, limited risk stratification tools, and challenges in tissue accessibility. Noninvasive screening strategies are scarce, and only 20% are diagnosed with resectable disease [1]. An ideal screening test would be a blood test that reliably detects a marker or markers specifically produced by PDAC cells at an early stage, prior to metastasis, that can survive hepatic filtration with a high sensitivity and specificity.

As *KRAS* mutations are found in over 90% of PDAC cases, the detection of *KRAS* mutations in ctDNA is a key target for screening assays [33,34]. Notably, *KRAS* mutations are early fundamental biological events in PDAC oncogenesis and may develop several years before clinical diagnosis, offering a potential window for early intervention. However, the pancreas drains primarily into the portal venous circulation, causing significant hepatic filtration (first-pass effect) of tumor DNA before reaching peripheral blood. This effect contributes to a low sensitivity of peripheral ctDNA assays in early stage PDAC. Studies have demonstrated higher ctDNA detection rates in portal blood compared with peripheral blood, particularly in early or low-volume disease [35,36]. A prospective study by Nitschke et al. analyzed ctDNA from both peripheral and portal venous blood in resectable PDAC patients [35] and found that *KRAS*-mutant ctDNA detection was significantly associated with shorter recurrence-free survival (*p* < 0.015). Notably, portal venous blood yielded a higher sensitivity for *KRAS*-mutant ctDNA detection and stronger prognostic significance than peripheral sampling [35]. These findings highlight a key limitation of using a ctDNA peripheral assay as a screening tool in PDAC, although they still reinforce the potential clinical value of postoperative ctDNA in detecting MRD and risk-stratifying patients for recurrence before radiographic evidence emerges [37,38,39,40].

The GRAIL’s Circulating Cell-Free Genome Atlas (CCGA) study enrolled over 15,000 participants to evaluate ctDNA-based multi-cancer early detection using a targeted methylation assay [41]. In its 2020 analysis, for PDAC, the sensitivity was 63% in Stage I and nearly 100% in Stage IV disease. While sensitivity remains a critical factor in screening, the high specificity of ctDNA is particularly valuable [41]. Accordingly, the American Society of Clinical Oncology and the College of American Pathologists opines that ctDNA is not yet validated for early detection or population screening, though it holds promise for treatment and MRD monitoring [42]. On the other hand, studies have shown that combining CA19-9, the only tumor marker validated to date in PDAC, with ctDNA (or other assays such as protein or methylation-based markers) improve early detection, signaling hope for the future [26,31,32].

### 2.3. Differentiating Benign and Pancreatic Cysts with Malignant Potential

In the field of gastrointestinal oncology, pancreatic cysts also present diagnostic and management challenges. While most cysts are benign, some can carry a malignant potential. Given the poor prognosis of PDAC, being diagnosed with a pancreatic cyst often triggers patient anxiety and can lead to costly, invasive, and sometimes unnecessary interventions. Plasma ctDNA, including *KRAS* mutations, may correlate with tumor burden, but its sensitivity for early or premalignant disease is low and not recommended for cyst evaluation or screening [26,43,44,45]. In contrast, cyst fluid DNA analysis, despite its low cellularity, can provide useful insights, including actionable mutations, and improve risk stratification when combined with imaging and clinical data [46,47,48].

### 2.4. Measuring Minimal Residual Disease

Multiple systematic reviews and metanalyses have consistently demonstrated that postoperative ctDNA positivity is a strong, independent predictor of overall survival in both curative and palliative cohorts of patients with solid tumors. In one metanalysis, encompassing over 3500 patients, ctDNA-positive individuals had a pooled hazard ratio of 7.27 for recurrence following curative-intent resection, with concordant findings across both tumor-informed and tumor-agonist assays [49,50,51]. Prospective and large cohort studies show that ctDNA can detect recurrence months before clinical or radiographic evidence, with lead times of 7–12 months [52,53]. ctDNA also outperforms traditional biomarkers like CEA [53,54,55,56]. Undetectable ctDNA postoperatively carries a high negative predictive value, supporting its role in adjuvant therapy de-escalation [57,58]. While most evidence is in CRC, similar applications are being explored in PDAC, with ongoing clinical trials assessing ctDNA-guided treatment strategies. Although other MRD biomarkers like CA19-9, circulating tumor cells (CTCs), and mRNA have been explored, ctDNA remains the most extensively studied. The optimal timing of ctDNA measurement varies across studies in the literature, however, typically, measurements are taken preoperatively, followed by the early postoperative period (generally 2 to 12 weeks after surgery), and then additionally for long-term follow up. Presence of detectable ctDNA is significantly associated with worse OS and PFS [59,60,61,62,63]. In resected PDAC patients, postoperative ctDNA positivity has demonstrated a high sensitivity (90%) and a high specificity (88%) for predicting disease recurrence [38]. Table 2 summarizes the clinical studies, systematic reviews, and meta-analyses evaluating MRD monitoring in PDAC. Current guidelines recommend adjuvant therapy for all resected PDAC patients who are fit; however, decisions about timing and regimen depend largely on recovery status and expected responsiveness. ctDNA trends from the neoadjuvant and early postoperative setting may help inform these decisions. Especially persistently elevated or rising postoperative ctDNA levels may support the need for aggressive adjuvant therapy.

### 2.5. ctDNA as a Tumor Marker to Monitor Chemotherapy Response

#### Monitoring Therapy in Neoadjuvant and Palliative Settings

Neoadjuvant chemotherapy is commonly used in resectable and borderline resectable PDAC with treatment response traditionally assessed by CT, PET/CT, or MRI, although these modalities can struggle to distinguish viable tumor from post-treatment inflammation or fibrosis. Advanced imaging methods like PET/MRI and diffusion-weighted MRI, along with radiomics, show promise, but are not yet standard and still require further validation [69,70,71].

ctDNA provides a complimentary, real-time measure of tumor burden and responsiveness. In a prospective cohort of localized PDAC, serial ctDNA analysis revealed that patients who had detectable ctDNA after neoadjuvant chemotherapy had a higher CA19-9 level along with worse PFS. Additionally, the presence of *KRAS* mutations in ctDNA at the time of diagnosis independently predicted inferior outcomes. ctDNA clearance during therapy was also associated with improved prognosis [19]. Another prospective study used digital droplet PCR and found that the clearance of *KRAS* mutations in ctDNA during the neoadjuvant therapy period was associated with significantly improved OS, however, persistent or new detectable ctDNA after treatment resulted in poor outcomes [72]. Postoperative ctDNA positivity is also a strong predictor of early relapse and decreased survival, especially when combined with elevated CA19-9 levels [73]. Longitudinal ctDNA profiling has demonstrated that changes in ctDNA allele correlate with clinical response and disease burden.

Serial monitoring of ctDNA during neoadjuvant therapy may provide an early indication of tumor response; declining levels are associated with radiographic regression and may help guide timing for surgical resection. For example, patients who show strong ctDNA clearance with less intensive regimens such as gemcitabine may avoid escalation to mFOLFIRINOX, particularly if the postoperative functional status is marginal. Conversely, lack of ctDNA clearance or rising levels during or after neoadjuvant therapy may prompt a change in adjuvant strategy. This additional insight may help guide both the treatment selection and prediction of neoadjuvant therapy response.

In advanced PDAC, ctDNA monitoring provides earlier and more sensitive detection of disease progression along with treatment response when compared with imaging and serum tumor markers. Multiple studies in advanced PDAC and other solid tumors have shown that dynamic changes in the level of ctDNA correlate with clinical outcomes and often precede radiologic evidence, up to a median of 19–23 days [14,74,75,76]. Quantitative ctDNA mirrored the disease state, with the disappearance of ctDNA after initial chemotherapy correlating with longer PFS [77]. In particular, in metastatic CRC, ctDNA was more sensitive than CEA for detecting disease progression and responded more rapidly to changes in tumor burden [78]. Similarly, these findings have also been reported in other solid tumors, where ctDNA use during therapy predicted the time to treatment failure and other clinical outcomes, often preceding clinical or radiographic signs of progression [75,79,80].

### 2.6. Patient Selection for Precision Medicine

Unresectable and metastatic PDACs account for nearly 80% of new PDAC diagnoses and present unique opportunities for precision medicine [81]. ctDNA serves as a noninvasive alternative for real-time genomic profiling, particularly when tumor tissue is limited or insufficient. In this setting, ctDNA has the potential to expand access to biomarker-driven treatments in PDAC by enabling real-time, noninvasive genomic profiling. ctDNA can be utilized to identify actionable mutations such as alterations in *KRAS*, *BRCA*, *EGFR*, *BRAF*, *PIK3CA*, which may inform targeted therapy selection. In a large cohort study, ctDNA identified relevant alterations for precision medicine in nearly half of the patients with advanced disease [82]. The strong agreement between ctDNA results and tissue mutation profiling results by NGS reinforces the utility of ctDNA guiding targeted therapies [83]. Furthermore, longitudinal tracking of ctDNA profiling can identify clonal evolution along with emerging resistance, allowing for a refined approach to precision medicine [84,85].

While most of the evidence comes from other related gastrointestinal malignancies, the findings are increasingly applicable to PDAC, where tissue samples are difficult to obtain. In a study evaluating advanced biliary tract cancers, ctDNA showed a high concordance (85%) with tissue based NGS and successfully identified actionable alterations. Some alterations like *FGFR2* fusions and *IDH1* mutations were reported with ctDNA while missing out on tissue analysis [86,87]. A recent prospective cohort study of 30 patients with resectable or borderline PDAC demonstrated the feasibility of the detection of pathogenic variants in baseline plasma using a tumor agnostic approach [88]. A large prospective study conducted by the National Center for Precision Medicine in France evaluated the utility of ctDNA sequencing across a cohort of 1772 patients. Their study provides compelling evidence that ctDNA profiling can facilitate timely and personalized treatment strategies, particularly when the tissue sample is limited or inaccessible [89]. In another retrospective cohort study of 259 inoperable PDAC patients, researchers evaluated the clinical potential of analyzing plasma-derived cfDNA using a two stage approach by combining droplet digital PCR (ddPCR) and targeted deep sequencing. *KRAS* mutations were detected by ddPCR in 58.9% of the cases, and subsequent cfDNA sequencing in a subset of 48 *KRAS*-mutant plasma samples identified potential actionable mutations in 29.2% of the patients. This demonstrates that ctDNA can uncover alterations beyond *KRAS*, expanding the therapeutic landscape for precision medicine [90].

## 3. Limitations and Challenges

Despite promising preliminary results, ctDNA faces several limitations that hinder routine clinical implementation. The current evidence indicates that in early stage PDAC, ctDNA concentrations are often below the threshold for detection, reducing its sensitivity for screening or early detection. This low sensitivity is due to several reasons. Tumor biology is different across patients, and not all tumors shed detectable levels of ctDNA into circulation [91,92,93]. An additional biological factor limiting ctDNA detection is hepatic filtration, especially in patients who have localized disease, as it can further reduce the detectability [5,94,95]. Furthermore, some rare driver mutations can be missed if they fall outside standard assay panels [42,96,97]. As a result, a negative ctDNA result cannot confidently exclude the presence of disease, especially in early stage PDAC.

In terms of measuring MRD, ctDNA positivity postoperatively clearly indicates a high risk of recurrence and poor prognosis. However, it remains uncertain how positive ctDNA results should be acted upon. Currently, no sufficient data are available to recommend intensification or the switching of adjuvant therapies in these patients. Further studies are needed to understand the best treatment strategies for these patients. Similarly, how we should use ctDNA in disease monitoring during neoadjuvant, adjuvant, and palliative chemotherapy is not clear at present. Although it is logical to use ctDNA in CA19-9 negative patients for disease monitoring, in those with detectable CA19-9, how best we should use ctDNA remains unanswered. Most studies that have attempted to answer these questions were exploratory in nature, and further rigorous validation studies will be needed before the role of ctDNA can be clearly defined in improving patient outcomes.

There also remains unresolved technical challenges that include inconsistent sensitivity across the different assays and discrepancies between the tissue-based NGS and ctDNA findings. An additional challenge is setting the appropriate variant allele frequency thresholds and distinguishing true mutations from background noise or low-level artifacts [98].

False positives from clonal hematopoiesis, the lack of assay standardization, variability in insurance coverage, high out-of-pocket costs for patients, and unclear reimbursement policies remain barriers to widespread adoption [99,100]. Reimbursement policies are inconsistent, and regulatory oversight is still evolving. A joint review from the American Society of Clinical Oncology and College of American Pathologists by Merker et al. emphasized the need for rigorous assay validation, clinical-context-specific interpretation, and harmonized standards before ctDNA can be fully integrated into routine care [42]. Clinical uncertainty persists in the world of ctDNA due to the lack of standardized algorithms to guide clinical decision-making based on ctDNA results outside of the context of clinical trials. Continued technological advances and regulatory guidance will be essential to overcome these challenges [99].

## 4. Discussion and Future Directions

Currently, ctDNA offers a unique and minimally invasive way to monitor tumor biology and molecular characteristics throughout all stages of PDAC. Its most established clinical applications are for monitoring for recurrence and guiding molecular targeted therapies, especially when sufficient tumor tissue is not available for genomic testing. However, its broad integration into areas such as early detection and treatment decisions regarding adjuvant therapy remains more nuanced, requiring more active investigation. Important considerations include how ctDNA can be effectively combined with imaging techniques and establish markers such as CA19-9, how low-level or equivocal ctDNA marker results should be interpreted, and ways to achieve consistent testing approaches across various institutions.

These limitations are beginning to be addressed by emerging strategies. These include integrating ctDNA with clinical history, radiological imaging, and traditional serum biomarkers, supported by artificial intelligence to create dynamic and personalized treatment plans [101,102,103,104]. As technology advances, the establishment of standardized protocols and clear regulatory approvals will be essential for widespread clinical adoptions. However, as ctDNA testing becomes more routine, ethical and practical issues are bound to arise. Some of the challenges include incidental findings (such as clonal hematopoiesis or germline mutations), interpreting uncertain results, and patient anxiety associated with a positive liquid biopsy without any radiographic correlation. Recent guidance emphasizes that the utility of ctDNA varies by disease subtype, suggesting that PDAC specific assay sensitivities and shedding thresholds may be required, rather than applying a generalized gastrointestinal cancer framework [105]. Furthermore, the cost effectiveness of ctDNA, particularly in early stage PDAC, is limited and remains essential for broader reimbursement and clinical acceptance.

ctDNA is also gaining attention as a valuable tool for detecting micrometastatic disease in resectable or localized PDAC. Despite absences in radiographic abnormalities, ctDNA positivity following neoadjuvant therapy or surgery may indicate residual subclinical disease, often seen in the peritoneum or liver that may evade conventional imaging. This insight can be utilized to justify intensified adjuvant therapy or surveillance. Additionally, ctDNA clearance may be used for treatment de-escalation, improving patient quality of life by potentially minimizing overtreatment. However, hepatic filtration can reduce the ctDNA detectability from peritoneal micrometastases. This emphasizes the necessity of a multimodal monitoring approach and presents a potential for future prospective clinical trials exploration.

**Figure 1:**
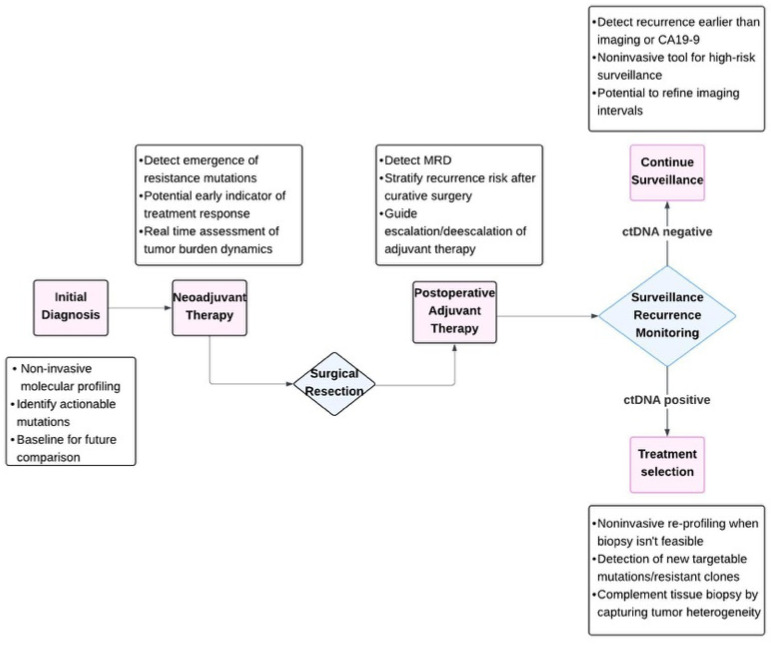
Utility of ctDNA through different phases of clinical care in PDAC.

We summarize the potential clinical utility of ctDNA in patients with PDAC in Figure 1. Currently, there are several clinical trials that are actively evaluating ctDNA in PDAC, particularly focusing on key applications such as early detection, MRD monitoring, and assessing treatment response [106,107,108,109,110]. In parallel, multiple studies have confirmed the diagnostic and prognostic value of CA19-9 as the most validated biomarker in PDAC, with consistent sensitivity and specificity reported across cohorts [27,28,29,30]. These findings reinforce CA19-9 as the benchmark serum marker while also discussing its limitations and need for complementary biomarker tools like ctDNA. Clinical trials that are evaluating ctDNA’s role in PDAC include early detection trials such as NCT03524677 and NCT04246203, which are evaluating mutation panels in high-risk populations [106,107,108]. In molecularly selected subgroups, trials such as APOLLO (NCT04858334) are evaluating ctDNA to guide targeted therapy choices [109]. On ClinicalTrials.gov, over 40 ctDNA-related PDAC studies are currently active. These include plasma-based genomic signature trials, surveillance studies to guide adjuvant therapy, and serial ctDNA tracking trials for resistance profiling [106,107,110]. Several studies are exploring the integration of ctDNA with imaging or radiomics into composite clinical prediction models [110].

Furthermore, ctDNA’s incorporation into clinical trials as an exploratory endpoint or stratification tool is increasingly being implemented. Although ctDNA is not yet validated as a surrogate marker for OS or PFS, it holds promise in early phase trials for monitoring treatment efficacy and early resistance [105]. These recommendations align with our proposal to expand ctDNA’s role within adaptive clinical trial designs that personalize treatment in real-time. Several prospective studies and meta-analyses reinforce the role of perioperative ctDNA as an independent predictor of recurrence and survival in PDAC. These studies underscore the prognostic value of ctDNA in postoperative settings, which supports its integration into future clinical trials [64,65,66,67,68].

As research progresses, positive trial outcomes will help facilitate the inclusion of ctDNA testing in clinical guidelines, standardize the use of ctDNA, and clarify reimbursement policies. Future directions involve integrating ctDNA assays with other innovative approaches such as methylation profiles, fragmentomics analysis, and AI-driven prediction models. Other uses for ctDNA include evaluating ctDNA as a predictive marker for immunotherapy response, tracking resistance in real-time, and assessing tumor heterogeneity across metastatic sites through a noninvasive approach. The hope is that with increasing implementation of ctDNA, we can use it to guide decisions to reduce therapy intensity in patients who have achieved sustained responses and track treatment effects in early phase clinical studies. Furthermore, identifying early signs of organ-specific recurrence (particularly the liver) is an ongoing interest. Nevertheless, these applications still need prospective validation. They, however, highlight ctDNA’s growing potential in precision oncology.

## 5. Conclusions

ctDNA holds significant promise in transforming the management of PDAC by enabling earlier detection, individualized treatment strategies, and improved clinical outcomes. Despite this potential, broader adoption in clinical practice will depend on ongoing advances in assay technology, integration with complementary biomarkers, and the establishment of standardized protocols. Addressing the challenges associated with low ctDNA shedding in early stage disease and tumor heterogeneity, along with assay standardization, is crucial. Additionally, assessing the cost-effectiveness and clinical impact of routine ctDNA implementation, especially in early detection or screening scenarios, remains essential. Prospective clinical trials are needed for validating ctDNA’s utility across various stages of PDAC care.

## Figures and Tables

**Table 1:** Sensitivity and specificity of circulating biomarkers in PDAC.

Biomarker	Disease Stage	Sensitivity (%)	Specificity (%)	Notes and References
CA 19-9	All stages	72–80	86–93	Best validated; sensitivity drops in Lewis antigen-negative patients; lower specificity in benign disease [26,27,28]
CA 19-9	Pre-diagnosis	50–64	99	Increases in CA19-9 up to two years to diagnosis; 64% sensitivity at 99% specificity to differentiate resectable PDAC from healthy controls; Sensitivity dropped in patients with benign pancreatic disease when specificity was kept at 99% [29,30]
ctDNA (*KRAS* mutations)	All stages	48–65	75–94	Sensitivity lower than CA19-9; specificity high; performance improves with stage [7,22,26,31]
ctDNA (*KRAS* mutations)	Stages I and II	48–50	94	Sensitivity limited in early stage; specificity remains high [7,31]
ctDNA	Locally advanced and metastatic	>75–94	94–99	Sensitivity increases with tumor burden; high specificity [7,20,22]
CA19-9 + ctDNA	All stages	78	91	Combination improves diagnostic accuracy over either alone [26,31,32]

**Table 2:** Studies that evaluated the value of ctDNA for MRD monitoring after surgery in PDAC.

Study	Type of Study	Patient Number	Timing of MRD Monitoring	Biomarkers Measured	Key Findings
Groot VP et al. [38]	Prospective single institution cohort study	59	Preop, immediate postop, serial follow-up	ctDNA (*KRAS* mutations)	Postop ctDNA positivity predicts recurrence with median lead time of 84 days, ctDNA levels dropped significantly after resection.
Yanala UR et al. [64]	Single institution case series and meta-analysis	171	End-of-treatment (after completion of all curative-intent surgery and chemotherapy), surveillance	ctDNA (tumor-informed NGS), CA19-9	End of treatment ctDNA positivity has high specificity and positive predictive value for recurrence and is associated with significantly worse recurrence free survival.
Lee B et al. [65]	Prospective multicenter biomarker trial	81	Preop, postop (timing not specified)	ctDNA (*KRAS* mutations), CA19-9	Postop ctDNA positivity is associated with 100% recurrence and poor OS, even with adjuvant chemotherapy.
Botta GP et al. [24]	Retrospective real-world data analysis	298	Periop (2–12 weeks postop), surveillance (>12 weeks postop or postadjuvant)	ctDNA (tumor informed NGS)	Positive ctDNA detection is significantly associated with shorter disease-free survival. Surveillance ctDNA is the most significant prognostic factor for recurrence.
Lee JS et al. [66]	Meta-analysis	375	Preop, postop (timing not specified)	ctDNA	Positive ctDNA is associated with poor overall survival (HR 3.66) and is also associated with a higher recurrence risk.
Vidal L et al. [67]	Meta-analysis	413	Preop, postop (timing not specified)	ctDNA, CTCs, mRNA	Perioperative ctDNA positivity is associated with worse prognosis. Surgical resection increases ctDNA clearance.
Hata T et al. [68]	Prospective single institution cohort study	66	Preop, immediate postop	ctDNA (*KRAS* mutations by droplet PCR)	Detectable postop ctDNA were more likely to develop hepatic recurrence. Preoperative ctDNA did not affect long term outcomes. Postoperative ctDNA has an independent recurrence risk.

## Abbreviations

The following abbreviations are used in this manuscript:

AI	Artificial intelligence
ALK	Anaplastic lymphoma kinase
BRCA1/2	Breast cancer gene 1 and 2
BRAF	B-Raf proto-oncogene
CA19-9	Carbohydrate antigen 19-9
CEA	Carcinoembryonic antigen
cfDNA	Cell-free DNA
CRC	Colorectal cancer
CT	Computed tomography
ctDNA	Circulating tumor DNA
ddPCR	Droplet digital polymerase chain reaction
EGFR	Epidermal growth factor receptor
FDA	U.S. Food and Drug Administration
FGFR2	Fibroblast growth factor receptor 2
IDH1	Isocitrate dehydrogenase 1
KRAS	Kirsten rat sarcoma viral oncogene
MRI	Magnetic resonance imaging
MRD	Minimal residual disease
NGS	Next-generation sequencing
OS	Overall survival
PDAC	Pancreatic ductal adenocarcinoma
PET	Positron emission tomography
PFS	Progression free survival (PFS)
